# Bolajoko Olusanya: personal challenges, public health

**DOI:** 10.2471/BLT.19.031019

**Published:** 2019-10-01

## Abstract

Bolajoko Olusanya talks to Sophie Cousins about her personal experience with hearing impairment and her efforts to advance paediatric hearing health in Nigeria.

SC: I understand your work has been inspired by your personal experience of congenital hearing disability. Is that correct?

BO: That’s right, I was born with mid-frequency hearing loss and struggled with inter-personal communication as a child without knowing what was wrong. In fact, I wasn’t diagnosed until I was 33 years old.

SC: How did that come about?

BO: I was doing a hearing test for a child with otitis media at a community centre for child development in East London and was surprised to find the child responding to sound levels that I couldn’t hear myself. After discharging the child, I immediately tested my own hearing. The audiogram showed a ‘cookie-bite’ mid-frequency bilateral hearing loss, which was subsequently confirmed by the centre audiologist.

SC: That must have been quite a revelation.

BO: It was. It was also something of a turning point for me. It brought back a lot of memories of my childhood including being ridiculed and taunted. I grew up an angry child from frequent and unjust punishment for not doing what I was told even though I always did what I heard. It really made me think, particularly about all the children with hearing loss who were going through similar struggles, but without the advantages that I had. I felt morally bound to do something for those children and decided to use all my education and training to draw attention to the challenges they and their families face. I started by conducting an audiological survey of children in Lagos schools in 1994.

SC: What did you find?

BO: Remarkably, I found that 13.9% of school entrants had some degree of hearing loss. Frankly it surprised me, but it was later corroborated by a more robust WHO-supported national hearing survey, which was carried out between 1999 and 2001 and reported a national prevalence of 13.4% among school children. My research showed not only the true prevalence of hearing loss among children in Nigeria, but the range of personal coping strategies they were using to be able to function and to avoid discrimination and stigma.

SC: Are discrimination and stigma significant issues in Nigeria?

BO: Yes, hearing impairment is associated with superstitions in many communities, which feed into both. However, the major reason for the issue’s neglect and, so to speak, ‘invisibility’ is the fact that it is not associated with significant mortality.

“[I] wanted to galvanise research that could translate into policy initiatives.”

SC: Can you explain what you mean by that?

BO: Nigeria, like most developing countries, is greatly influenced by global health priorities and funding programmes, which tend to prioritise reducing mortality or ensuring survival rather than supporting or promoting well-being.

SC: Is that why you set up your different non-governmental organisations – to address that neglect?

BO: Certainly, that was part of it, but I also wanted to galvanise research that could translate into policy initiatives for the public good. Basically, after I completed my studies I was faced with a choice: I could pursue a professorial career in my university, which would of course mean working within the administrative constraints of academia or become a social entrepreneur with unconventional community engagement on issues that I was passionate about. I chose the latter and set up Hearing International Nigeria (HING), which was officially launched in 1999 by Professor Peter Alberti, the then Secretary-General of the International Federation of Otorhinolaryngological Societies. I also received a great deal of support from Professor Robert Ruben, the Editor-in-Chief of the International Journal of Pediatric Otorhinolaryngology.

SC: What was the main focus of your work with HING?

BO: I worked on the development of effective child health policy based on the research I had done on hearing loss prevalence. I conducted a new study of two schools for deaf children with the aim of determining modifiable causes or risk factors for hearing impairment. I found that a high proportion of the children had congenital hearing loss, and the parents only provided very limited insights on the underlying causes, so it was something of a dead end. To gain more insight into audiological medicine I committed to pursuing a PhD in Child Health at UCL with Professor Linda Luxon. I formed the Nigerian Dyslexia Association which was combined with HING into the Centre for Healthy Start Initiative, the umbrella nongovernmental organization for all my research and advocacy activities, in 2011.

SC: What inspired you to start an organisation focused on dyslexia?

BO: I could see that there was a lack of awareness about dyslexia in Nigeria and a lack of services in support of people living with the condition. However, as with the hearing impairment issue, my personal experience was a key motivator. My husband is dyslexic, as is one of my children. The NDA creates awareness and training for teachers on dyslexia in mainstream schools and advocates for support for children with learning disabilities. Inspired by my husband’s success story as an accomplished banker, we have been able to advocate and to support awareness programmes and training for teachers. I am pleased to report that a number of children who have been helped by the programme have gone on to university, including my son who has a master’s degree in software engineering from the UK and is a successful entrepreneur in Nigeria.

SC: You also worked on developing hospital and community-based infant hearing screening programmes in Nigeria. Can you talk about that?

BO: To get a clearer picture of the epidemiology of hearing loss, to understand the drivers of incidence and potential public approaches to prevention, treatment and support, you need to implement effective screening programmes. Because of the high proportion of babies not born in hospitals, I considered it essential to have a community-based universal infant hearing screening programme, to compliment the hospital-based programme. The programme was launched in 2005. About 12 000 mother-infant pairs were enrolled – 5 000 for the hospital-based programme and 7 000 for the community-based programme. The programmes constituted a very effective platform for investigating the profile of hearing loss in newborns and any related maternal factors in great detail and were the basis for numerous novel findings.

“Opportunities are emerging […] for collaboration across disciplines.”

SC: Can you be specific?

BO: One key finding was the contribution of severe neonatal jaundice to the incidence of hearing loss. This finding led to several interventions to reduce the incidence of severe jaundice in the two centres where the newborn hearing screening programmes were conducted. For instance, we were able to install a simple low-cost technology that uses filtered sunlight for treating children with jaundice. From its inception in 2011, the centre has been providing free screening and treatment for jaundice for all newborns at two state hospitals as part of its community engagement to reduce the incidence of bilirubin-induced disabilities. We were also able to show how routine immunisation clinics could deliver essential child health services such as newborn hearing screening and baseline anthropometry for growth monitoring to infants not born in hospitals. Finally, we were able to make a number of observations regarding maternal health, mode of delivery, health-seeking behaviour, immunisation uptake and undernutrition. For example, because we enrolled participants under the hospital-based programme during maternal visits to antenatal clinics, we were able to track the incidence of stillbirths and related maternal risk factors. Unfortunately, both programmes were terminated in 2008 due to funding constraints and the lack of government-backed public health policy at the state and federal levels.

SC: You have talked about the importance of cross-disciplinary collaboration for the prevention and management of early childhood disabilities. To what extent is this approach being used in Sub-Saharan Africa?

BO: Children living with disabilities need the support of a wide range of specialists, including paediatricians, psychologists, neurologists, audiologist and otorhinolaryngologists – to name just a few. Establishing and coordinating the services from these various disciplines efficiently for the benefit of the patient is often a challenge. The need for coordination and collaboration is even greater in sub-Saharan Africa where there is an acute shortage of skilled personnel. Working with children and their families, I believe that paediatricians or other childcare specialists are in a better position to coordinate and lead such collaboration. Unfortunately, the implementation of this approach has so far been hindered by the lack of policy initiatives at the national or global level to address early childhood disabilities. It is only now that opportunities are emerging through the sustainable development goals for collaboration across disciplines. 

